# Antibodies against the Majority Subunit (PilA) of the Type IV Pilus of Nontypeable Haemophilus influenzae Disperse Moraxella catarrhalis from a Dual-Species Biofilm

**DOI:** 10.1128/mBio.02423-18

**Published:** 2018-12-11

**Authors:** Elaine M. Mokrzan, Laura A. Novotny, Kenneth L. Brockman, Lauren O. Bakaletz

**Affiliations:** a Center for Microbial Pathogenesis, The Research Institute at Nationwide Children’s Hospital, Columbus, Ohio, USA; b Department of Pediatrics, The Ohio State University College of Medicine, Columbus, Ohio, USA; GSK Vaccines

**Keywords:** LuxS, OMP P5, otitis media, quorum sensing, vaccine

## Abstract

Otitis media (OM) is often polymicrobial, with nontypeable Haemophilus influenzae (NTHI) and Moraxella catarrhalis (Mcat) frequently cocultured from clinical specimens. Bacterial biofilms in the middle ear contribute to the chronicity and recurrence of OM; therefore, strategies to disrupt biofilms are needed. We have focused our vaccine development efforts on the majority subunit of NTHI type IV pili, PilA. Antibodies against a recombinant, soluble form of PilA (rsPilA) both disrupt and prevent the formation of NTHI biofilms *in vitro.* Moreover, immunization with rsPilA prevents and resolves NTHI-induced experimental OM. Here, we show that antibodies against rsPilA also prevent and disrupt polymicrobial biofilms. Dual-species biofilms formed by NTHI and Mcat at temperatures that mimic the human nasopharynx (34°C) or middle ear (37°C) were exposed to antiserum against either rsPilA or the OMP P5 adhesin of NTHI. NTHI+Mcat biofilm formation was significantly inhibited by antiserum directed against both adhesin proteins at either temperature. However, only anti-rsPilA disrupted NTHI+Mcat preestablished biofilms at either temperature and actively dispersed both NTHI and Mcat via interspecies quorum signaling. Newly released NTHI and Mcat were significantly more susceptible to killing by antibiotics. Taken together, these results revealed new opportunities for treatment of biofilm-associated diseases via a strategy that combines vaccine-induced antibody-mediated biofilm dispersal with traditional antibiotics, at a significantly reduced dosage to exploit the newly released, antibiotic-sensitive phenotype. Combined, our data strongly support the utility of rsPilA both as a preventative and as a therapeutic vaccine antigen for polymicrobial OM due to NTHI and Mcat.

## Abstract (precis)

Middle ear infections (or otitis media [OM]) are highly prevalent among children worldwide and present a tremendous socioeconomic challenge for health care systems. More importantly, this disease diminishes the quality of life of young children. OM is often chronic and recurrent, due to the presence of highly antibiotic-resistant communities of bacteria (called biofilms) that persist within the middle ear space. To combat these recalcitrant infections, new and powerful biofilm-directed approaches are needed. Here, we describe the ability to disrupt a biofilm formed by the two most common bacteria that cause chronic and recurrent OM in children, via an approach that combines the power of vaccines with that of traditional antibiotics. An outcome of this strategy is that antibiotics can more easily kill the bacteria that our vaccine-induced antibodies have released from the biofilm. We believe that this approach holds great promise for both the prevention and treatment of OM.

## INTRODUCTION

Otitis media (OM) is the most common bacterial disease of childhood. By age 3, most children worldwide have had at least one episode of OM (1), and by 5 years of age up to 65% have experienced recurrent OM (2). The chronic and recurrent nature of OM is largely due to the presence of bacterial biofilms in the normally sterile middle ear space. Bacteria within a biofilm are highly resistant to antibiotics and recalcitrant to immune effectors, which makes chronic and recurrent OM extremely difficult to treat with standard antibiotic regimens.

Nontypeable Haemophilus influenzae (NTHI) is the predominant pathogen of chronic and recurrent OM, as well as OM that fails antibiotic treatment (3). Although NTHI is a commensal of the human nasopharynx, when the immune response and/or the normal protective mechanisms of the upper respiratory tract is compromised, NTHI can ascend the Eustachian tube to gain access to the middle ear and cause disease, which is complicated by the ability of NTHI to rapidly establish a biofilm. NTHI biofilms are integral to the pathogenesis of many respiratory tract infections, which include OM, chronic rhinosinusitis, bronchitis, community-associated pneumonia, and exacerbations of both cystic fibrosis and chronic obstructive pulmonary disease (4). Because bacterial colonization of the nasopharynx serves as the reservoir for NTHI that induces these diseases, mechanisms that mediate NTHI adherence, long-term colonization, or biofilm formation are strategic targets for disease prevention and/or treatment. Type IV pili (Tfp) are essential for NTHI adherence, biofilm formation, twitching motility, and competence (5–8), and as such, are important virulence determinants. Thereby, we targeted the major protein subunit of Tfp, PilA, as a vaccine candidate for diseases due to NTHI, which include OM. Antiserum against a recombinant soluble form of PilA (rsPilA) inhibits NTHI biofilm formation *in vitro* (9, 10) and prevents the onset of NTHI-induced OM in a chinchilla model (11). In addition, antibodies against rsPilA disrupt established NTHI biofilms both *in vitro* and *in vivo* (9–12). Hence, immunization with rsPilA holds great promise for the prevention and the therapeutic resolution of OM and many other NTHI-induced diseases.

In this study, we began to address the potential utility of this Tfp-targeted approach against more complex OM wherein NTHI coinfects with another otopathogen, Moraxella catarrhalis (Mcat), as these two species are often detected together in OM. Prior studies showed that 44% of patients with acute OM (13) and 33% of children with recurrent OM (14) had both NTHI and Mcat in their middle ear fluids. Unlike NTHI, which is often isolated as the sole pathogen, Mcat is most common in polymicrobial OM (13, 15, 16), and multiple studies show that NTHI enhances the ability of Mcat to colonize the nasopharynx (17) or to infect the middle ear (18, 19). Together, these facts suggest a symbiotic relationship between NTHI and Mcat that facilitates polymicrobial OM. Thus, it has been speculated that vaccine strategies that are specifically effective against NTHI-induced OM might also mediate a collateral, albeit indirect, benefit in terms of prevention or therapeutic resolution of polymicrobial OM.

Here, we explored the potential of rsPilA as a candidate vaccine antigen against polymicrobial OM due to NTHI and Mcat. Our results revealed a novel opportunity to prevent and/or resolve these complex infections with a synergistic approach that includes immunization with rsPilA in combination with use of a reduced dosage of the appropriate antibiotic, a strategy that has been referred to as “our best shot at combating drug-resistant microbes” (20).

## RESULTS

### NTHI surrounded Mcat-dense areas within dual-species biofilms.

**FIG 1:**
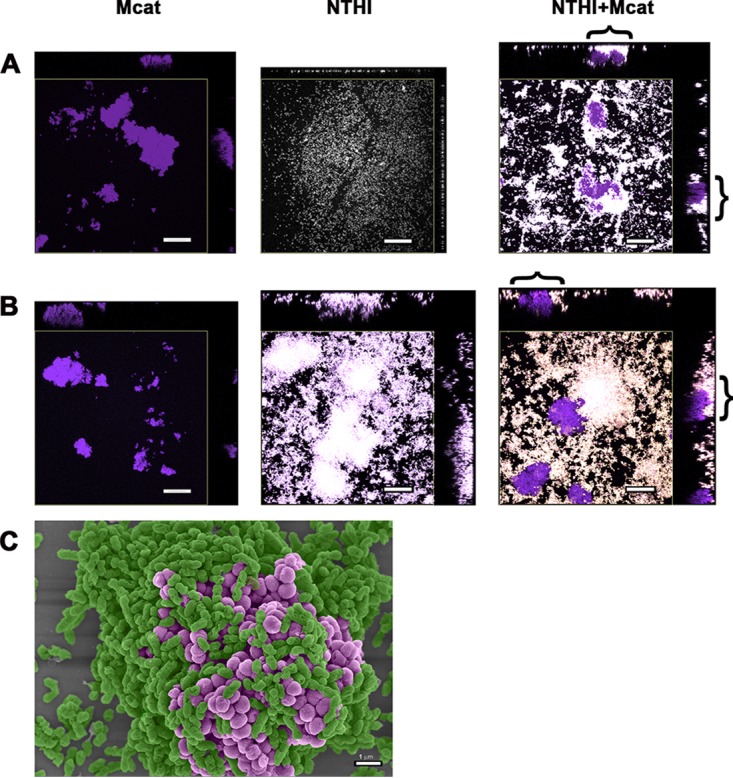
NTHI encircled and underlay Mcat within a dual-species biofilm. Orthogonal views of 16-h biofilms formed by NTHI (pseudocolored white), Mcat *(*pseudocolored purple), or a 1:1 mix of the two captured by CSLM. The central region of each image is a top-down view that depicts total biomass, whereas orthogonal views along the top and right margins show a single plane along the *x* and *y* axis. (A) At 34°C, Mcat biofilms comprised dense, compact towers with large intervening water channels (left image), whereas NTHI displayed minimal organization (center image). In NTHI+Mcat biofilms formed at 34°C, NTHI surrounded and underlay the Mcat-dense regions of the biofilm (right image). (B) At 37°C, Mcat biofilms resembled those formed at 34°C (left image). NTHI biofilms developed compact towers and intervening water channels (center image). NTHI+Mcat biofilms formed at 37°C incorporated NTHI towers which often encompassed and underlay Mcat aggregates (right image). Brackets indicate regions of NTHI surrounding Mcat in dual-species biofilms as examples. Scale bars indicate 20 µm. (C) Scanning electron micrograph showing a top-down view of NTHI+Mcat biofilm formed at 37°C for 16 h. NTHI (pseudocolored green) closely surrounded and underlay the Mcat (pseudocolored purple) within the tower. Scale bar indicates 1 µm.

To examine the spatial relationship between NTHI and Mcat during biofilm formation *in vitro*, dual-species biofilms were established at 34°C, the temperature typical of the human nasopharynx (21). After 16 h, Mcat-only biofilms were comprised of dense, isolated towers (Fig. 1A, left). In contrast, NTHI biofilms grown under the same conditions displayed minimal organization and tower formation (Fig. 1A, middle). However, a unique biofilm architecture was observed upon coculture. Whereas Mcat again formed dense, isolated aggregate-like towers, NTHI covered more of the substratum, and now appeared more dense in areas that both underlay and encircled the Mcat aggregates (Fig. 1A, right image; see bracketed areas of orthogonal view).

Next we examined single- and dual-species biofilms formed at 37°C, the temperature of the human middle ear. After 16 h, Mcat-only biofilms resembled those formed at 34°C, with densely packed, isolated towers (compare left image in Fig. 1B with left image in Fig. 1A). As anticipated based on many of our previous studies, when incubated at 37°C, NTHI biofilms had characteristic towers and water channels (Fig. 1B, middle) (9). Coculture of NTHI+Mcat at 37°C also resulted in biofilms wherein dense aggregates of Mcat were underlain and closely surrounded by NTHI. Mcat clusters were often observed within NTHI towers (note bracketed regions of Fig. 1B, right, orthogonal views). The close physical association between NTHI and Mcat within the dual-species biofilms was even more apparent by scanning electron microscopy of NTHI+Mcat biofilms (Fig. 1C), wherein NTHI (green) encompassed the Mcat (purple). As previous work demonstrates that NTHI-only biofilms are significantly disrupted upon incubation with anti-rsPilA (9, 10), we wondered if a similar outcome would result in the context of a dual-species biofilm, and moreover, whether the close association between biofilm-resident NTHI and Mcat shown here would result in exposure of the associated Mcat to host immune defenses, or perhaps also be physically disrupted from the biofilm.

### NTHI PilA was expressed in biofilms formed by NTHI+Mcat.

**FIG 2:**
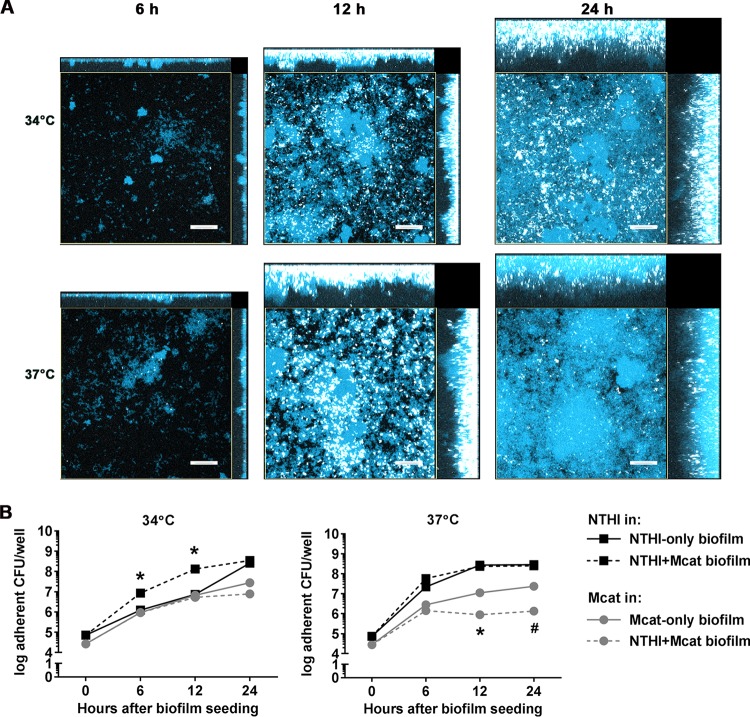
NTHI Tfp were expressed in dual-species biofilms formed at 34°C and 37°C. NTHI+Mcat biofilms were formed with a 1:1 mix of Mcat and NTHI:*pilA-*GFP. To visualize total biomass, biofilms were counterstained with FM4-64 (pseudocolored blue). (A) In biofilms formed at 34°C, NTHI *pilA* promoter activity was observed at 12 h in culture and increased through 24 h. In biofilms formed at 37°C, NTHI *pilA* promoter activity peaked at 12 h. (B) Growth of NTHI and Mcat within monospecies or dual-species biofilms. Growth of NTHI between 6 and 12 h during biofilm formation was significantly greater in dual-species versus NTHI-only biofilms at 34°C, whereas Mcat growth remained constant. At 37°C NTHI growth was similar in NTHI-only and dual-species biofilms; however, Mcat growth was diminished in the presence of NTHI. Scale bars indicate 20 µm. *, *P ≤ *0.0001; #, *P = *0.001, Student’s *t* test.

We previously showed that expression of *pilA* (and by inference Tfp) in NTHI-only biofilms is influenced by environmental cues such as the relative temperature of the middle ear (37°C) versus that of the nasopharynx (34°C) (9). To now confirm that NTHI expressed *pilA* when resident within a dual-species biofilm, we examined NTHI *pilA* promoter activity in NTHI+Mcat biofilms formed at either 37°C or 34°C *in vitro*. (9) Whereas in previous work we observed maximal *pilA* promoter in NTHI-only biofilms after 24 h in culture at 34°C (9), when grown with Mcat, substantial *pilA* promoter activity was observed after only 12 h (Fig. 2A, top row). In NTHI+Mcat biofilms formed at 37°C, the temporal and spatial patterns of *pilA* promoter activity were similar to those in NTHI-only biofilms when grown at 37°C, with maximal expression at 12 h in culture (Fig. 2A, bottom row). Expression of *pilA*, as assessed by quantitative RT-PCR, was 1.4-fold greater in NTHI+Mcat biofilms than in NTHI-only biofilms when formed at 34°C for 12 h (see Fig. S1 in the supplemental material). In contrast, expression of *ompP5*, which encodes the NTHI OMP P5 adhesin, remained unchanged.

10.1128/mBio.02423-18.1 FIG S1 Expression of NTHI *pilA* and NTHI *ompP5* in NTHI+Mcat biofilms relative to expression in NTHI-only biofilms. Expression of NTHI *pilA* was increased 1.4-fold in dual-species versus NTHI-only biofilms formed under the same conditions. In contrast, NTHI *ompP5* expression in NTHI+Mcat versus NTHI-only biofilms was similar. Biofilms grown for 12 h at 34°C were washed once with saline and collected with TRIzol reagent (Ambion; Thermo Fisher) by repeated forceful pipetting. RNA was quantified as reported previously (9). Results were reported as relative gene expression in NTHI+Mcat compared to NTHI-only biofilms by the comparative (ΔΔ*C_*T*_*) method. Download FIG S1, TIF file, 1.7 MB. Copyright © 2018 Mokrzan et al. 2018 Mokrzan et al. This content is distributed under the terms of the Creative Commons Attribution 4.0 International license.

Because the images of NTHI+Mcat biofilms formed at 34°C appeared to show increased numbers of NTHI compared to NTHI-only biofilms (Fig. 1A, top row), we quantitated NTHI and Mcat in mono- and dual-species biofilms over time. As anticipated, after 6 and 12 h of culture there were significantly more NTHI in dual-species than in NTHI-only biofilms at 34°C, whereas there was no difference in CFU of Mcat in single- versus dual-species biofilms when grown at this temperature (Fig. 2B). Conversely, in dual-species biofilms grown at 37°C, the numbers of NTHI were similar in NTHI+Mcat versus NTHI-only biofilms; however significantly fewer Mcat bacteria were present in NTHI+Mcat versus Mcat-only biofilms (Fig. 2B). Taken together, these data demonstrated that NTHI *pilA* was indeed expressed in NTHI+Mcat biofilms incubated at either 34°C or 37°C. Importantly, at the cooler temperature of the human nasopharynx, NTHI growth and *pilA* promoter activity were accelerated by the presence of Mcat within the biofilm. Moreover, these effects were specific to the biofilm state, since we found no significant differences in NTHI growth or *pilA* promoter activity when NTHI was grown at 34°C with or without Mcat in broth culture (Fig. S2).

10.1128/mBio.02423-18.2 FIG S2 NTHI growth and *pilA* expression at 34°C in planktonic culture were not affected by the presence of Mcat. (A) NTHI:*ompP2*-GFP, a strain which constitutively expresses GFP, was grown in broth with or without Mcat (1:1 ratio of NTHI to Mcat) at 34°C. NTHI growth, as estimated by change in GFP-specific fluorescence intensity over time, was not affected by the presence of Mcat. (B) NTHI:*pilA*-GFP strain grown in planktonic culture at 34°C was monitored for change in fluorescence intensity over time to evaluate NTHI *pilA* promoter activity during growth with or without Mcat. The ratio of fluorescence intensity due to *pilA/ompP2* promoter activities (see panel A) was used to normalize *pilA* promoter activity to cell number and revealed that the fluorescence ratio was similar during NTHI planktonic growth in the presence or absence of Mcat (1:1 ratio NTHI to Mcat). Together these data demonstrated that NTHI growth and *pilA* promoter activity in broth culture at 34°C were similar whether NTHI was grown alone or cocultured with Mcat. Download FIG S2, TIF file, 3.5 MB. Copyright © 2018 Mokrzan et al. 2018 Mokrzan et al. This content is distributed under the terms of the Creative Commons Attribution 4.0 International license.

### Antibodies against rsPilA or OMP P5 inhibited the formation of NTHI+Mcat biofilms.

NTHI Tfp play an important role in twitching motility and adherence, and antibodies against rsPilA inhibit NTHI adherence to both abiotic surfaces and epithelial cells (9, 22). Having confirmed expression of NTHI *pilA* and by inference Tfp by NTHI in NTHI+Mcat biofilms formed at both 34°C and 37°C, we hypothesized that exposure to anti-rsPilA would limit the formation of NTHI+Mcat dual-species biofilms given that NTHI both surrounded and underlay towers of Mcat biofilms in these cultures. To test this, NTHI+Mcat or Mcat-only biofilms were exposed to anti-rsPilA 1 h after seeding. Four hours later, antiserum treatment was removed and biofilms were incubated for an additional 16 h. We also tested antiserum against OMP P5, another critical NTHI adhesin that is important for adherence and colonization (12, 23), and antiserum against chimV4, a recombinant chimeric antigen comprised of protective epitopes from both OMP P5 and PilA (10, 12). Previous work showed that each of these antisera significantly inhibits the formation of NTHI-only biofilms (9).

**FIG 3:**
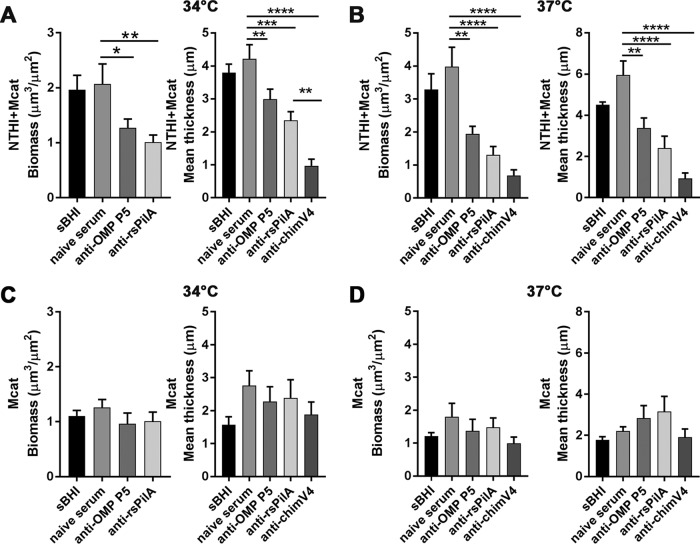
Antibodies against rsPilA or OMP P5 inhibited the formation of NTHI+Mcat biofilms. One hour after seeding, NTHI and Mcat were exposed to anti-rsPilA, anti-OMP P5, or anti-chimV4 or maintained in medium (sBHI). After 4 h, treatments were removed. After an additional 16 h of incubation, COMSTAT2 analysis of NTHI+Mcat biofilms formed at 34°C (A) or 37°C (B) showed significantly less biomass and reduced mean thickness in biofilms exposed to anti-rsPilA or anti-OMP P5 compared to biofilms incubated with naive rabbit serum as a negative control. The greatest inhibition of biofilm formation was due to antiserum against chimV4, which indicated an additive effect when both Tfp and OMP P5 are targeted, as by design. In contrast, there was no effect of any antisera tested against biofilm formation by Mcat alone at either 34°C (C) or 37°C (D). Data represent mean ± SEM of 3 (37°C) or 4 (34°C) experiments performed in duplicate. Statistical significance was determined by one-way analysis of variance with the Holm-Sidak correction. *, *P ≤ *0.05; **, *P ≤ *0.01; ***, *P ≤ *0.001; ****, *P ≤ *0.0001.

We found that NTHI+Mcat biofilm biomass and mean thickness were significantly reduced when exposed to anti-rsPilA serum shortly after seeding, compared to naive serum, at both 34°C and 37°C (Fig. 3A and B, respectively). NTHI+Mcat biofilms exposed to anti-OMP P5 serum were also significantly reduced compared to those incubated with naive serum, at either temperature. Biofilms exposed to anti-chimV4 shortly after seeding were the most inhibited, consistent with the anticipated additive effect achieved when both Tfp and OMP P5 are simultaneously targeted (Fig. 3A and B).

In contrast, there was no significant effect on Mcat-only biofilm formation regardless of whether these biofilms were exposed to anti-OMP P5, anti-rsPilA, or anti-chimV4 shortly after seeding (Fig. 3C and D). This result was expected given the specificity of these antibodies for NTHI proteins. Together, these results demonstrated that antisera against NTHI PilA and/or OMP P5 prevented the formation of dual-species biofilms, likely due to the fact that within a dual-species biofilm, Mcat is closely associated with and may be anchored to underlying NTHI.

### Antibodies against rsPilA disrupted established NTHI+Mcat biofilms.

**FIG 4:**
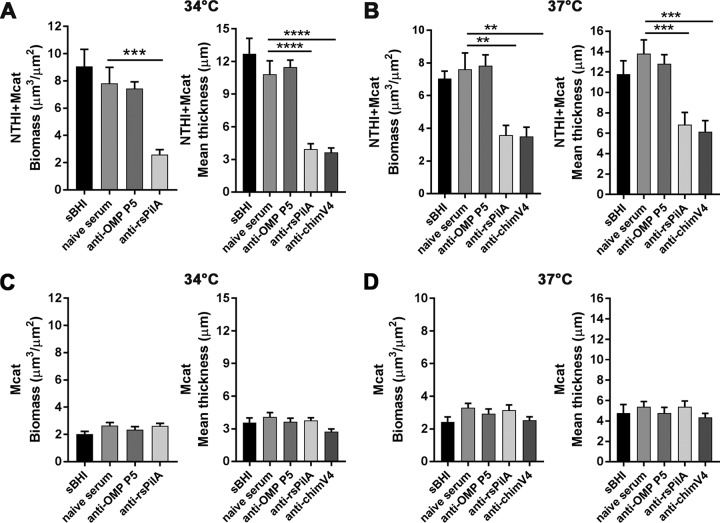
Antibodies that targeted NTHI PilA disrupted established NTHI+Mcat biofilms. Biofilms were established for 24 h; then exposed to anti-OMP P5, anti-rsPilA, or anti-chimV4 or maintained in medium (sBHI) for 16 h; and then visualized by CSLM. COMSTAT2 analysis of NTHI+Mcat biofilms formed at 34°C (A) or 37°C (B) revealed a significant reduction in biomass and mean thickness after exposure to anti-rsPilA or anti-chimV4, but not anti-OMP P5 or naive serum. In contrast, biofilms formed by Mcat alone showed no significant effect of any antiserum on biomass or mean thickness at either 34°C (C) or 37°C (D). These results demonstrated that NTHI+Mcat biofilms formed at either 34°C or 37°C were disrupted by antisera that target the NTHI Tfp. Data represent mean ± SEM from 3 experiments performed in duplicate. Statistical significance was determined by one-way analysis of variance with the Holm-Sidak correction. **, *P ≤ *0.01; ***, *P ≤ *0.001; ****, *P ≤ *0.0001.

Whereas most antibodies against NTHI outer membrane proteins are ineffective in disrupting preformed NTHI biofilms, antibodies against rsPilA are significantly disruptive (9, 10). Collectively, and based on our data to this juncture, we hypothesized that anti-rsPilA-induced dispersal of NTHI within a dual-species biofilm might also result in a collateral disruption of the closely physically associated Mcat. To this end, we established biofilms with either NTHI+Mcat or Mcat only for 24 h and then exposed them to anti-OMP P5, anti-rsPilA, or anti-chimV4 for an additional 16 h. As seen in Fig. 4, the biomass and mean thickness of established NTHI+Mcat biofilms exposed to anti-rsPilA or anti-chimV4 were significantly less than those of biofilms exposed to naive serum at both 34°C and 37°C (Fig. 4A and B). In contrast, anti-OMP P5 did not disrupt established NTHI+Mcat biofilms, an outcome that was not unexpected as this observation was consistent with previous results for NTHI-only biofilms (9). However, and also as expected, preformed Mcat only biofilms were unaffected by any of the NTHI-specific antisera tested, at either temperature (Fig. 4C and D). Together these results demonstrated that antisera that target NTHI PilA disrupted preestablished NTHI+Mcat biofilms, with significantly reduced overall biomass and thickness as the outcome. The latter result indicated a potential collateral, but indirect, effect on the cocultured Mcat.

### Anti-rsPilA induced the release of both NTHI and Mcat from dual-species biofilms.

**FIG 5:**
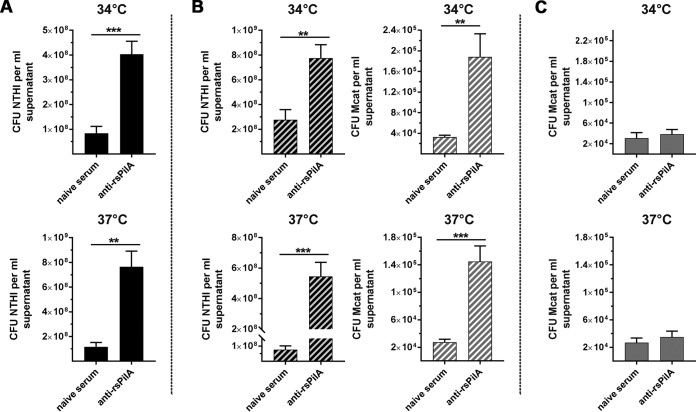
Anti-rsPilA induced the dispersal of both NTHI and Mcat from a dual-species biofilm. Biofilms were established overnight at 34°C or 37°C and treated with IgG-enriched anti-rsPilA or naive serum to achieve maximal dispersion (see Fig. S3 for timing of dispersal). (A) Significantly more NTHI was dispersed into the supernatant of NTHI-only biofilms exposed to anti-rsPilA compared to those exposed naive serum, at both 34°C and 37°C. (B) Significantly more NTHI (black striped bars) and Mcat (gray striped bars) were dispersed into the supernatant from NTHI+Mcat biofilms exposed to anti-rsPilA compared to biofilms treated with naive serum, at both 34°C and 37°C. (C) In contrast, no significant dispersal was observed when Mcat-only biofilms were exposed to anti-rsPilA compared to naive serum, at either 34°C or 37°C. Together these results demonstrated that treatment with anti-rsPilA induced the dispersal of both NTHI and Mcat from dual-species biofilms formed at 34°C or 37°C. Data represent mean ± SEM from 3 to 4 experiments performed in duplicate. Statistical significance was determined by Student’s *t* test. **, *P ≤ *0.01; ***, *P ≤ *0.001.

As anti-rsPilA induces active dispersal of NTHI from established biofilms (10), we next examined whether the decrease in biofilm biomass mediated by anti-rsPilA exposure (Fig. 4) reflected the release of both NTHI and Mcat from the dual-species biofilms. Thus, NTHI-only or NTHI+Mcat biofilms were incubated with anti-rsPilA, supernatants were collected at the time determined of peak dispersal (Fig. S3), and Mcat and NTHI were individually quantified. As expected, supernatants from NTHI-only biofilms formed at either 34°C or 37°C and then exposed to anti-rsPilA contained significantly more NTHI bacteria than supernatants from biofilms exposed to naive serum as a negative control (Fig. 5A). Intriguingly, supernatants from anti-rsPilA-treated NTHI+Mcat biofilms formed at either 34°C or 37°C contained significantly more of both NTHI and Mcat than did negative-control serum (Fig. 5B). We confirmed that this result was not due to a direct effect of anti-rsPilA on Mcat, since Mcat-only biofilms exposed to anti-rsPilA at either 34°C or 37°C showed no evidence of dispersal (Fig. 5C). Collectively, these data showed that antisera directed against rsPilA induced dispersal of both NTHI and Mcat from dual-species biofilms formed at the temperatures representative of both the human nasopharynx and middle ear.

10.1128/mBio.02423-18.3 FIG S3 Anti-rsPilA-mediated dispersion was influenced by temperature and the presence of Mcat. Optical density (OD_490_) of supernatants following exposure of 16-h biofilms formed at 34°C or 37°C to sBHI (open bars), 11 µg of IgG-enriched anti-rsPilA (gray bars), or naive serum (black bars). (A) For NTHI-only biofilms, a significant increase in OD_490_ was detected 8 h after anti-rsPilA exposure of NTHI-only biofilms formed at 34°C or 6 h after exposure of NTHI-only biofilms formed at 37°C. (B) For NTHI+Mcat biofilms, a significant increase in OD_490_ was detected 7 h after anti-rsPilA exposure at 34°C and 6 h after exposure at 37°C. These results indicated that biofilm dispersal induced by anti-rsPilA was influenced by temperature (likely a reflection of enhanced growth of NTHI at 37°C) and/or the presence of Mcat in the biofilm. Data represent mean ± SEM of 3 experiments performed in duplicate. Statistical significance was determined by one-way analysis of variance with the Holm-Sidak correction. Bars indicate *P ≤ *0.0001. Download FIG S3, TIF file, 2.9 MB. Copyright © 2018 Mokrzan et al. 2018 Mokrzan et al. This content is distributed under the terms of the Creative Commons Attribution 4.0 International license.

### Mcat was dispersed from NTHI+Mcat biofilms in response to AI-2 produced by NTHI.

Since anti-rsPilA neither inhibited the formation of Mcat-only biofilms (Fig. 3) nor disrupted those that were already formed (Fig. 4), we reasoned that Mcat dispersal likely occurred via an indirect mechanism. We previously demonstrated that anti-rsPilA induces an NTHI quorum-signaling event in which AI-2 is released in conjunction with NTHI dispersal from the biofilm (10). Armbruster et al. (19) showed that exogenous AI-2 increases the growth and antibiotic resistance of Mcat during NTHI+Mcat biofilm formation *in vitro*; i.e., Mcat can “eavesdrop” on AI-2 expressed by another microbe. Therefore, we hypothesized that AI-2 produced by NTHI after exposure to anti-rsPilA was detected by Mcat within the dual-species biofilm and thereby mediated Mcat dispersal.

To test this hypothesis, we established dual-species biofilms wherein Mcat was cocultured with either the NTHI parent strain, an NTHI mutant unable to produce AI-2 (Δ*luxS*), or a complemented Δ*luxS* strain for 16 h at 37°C followed by incubation with anti-rsPilA or naive serum for an additional 6 h. There was no difference in CFU of NTHI or Mcat in the supernatants collected from Mcat+NTHI Δ*luxS* biofilms exposed to anti-rsPilA versus naive serum; therefore, these bacteria were not dispersed (Fig. S4A and B). In contrast, CFU of both NTHI and Mcat were significantly greater in supernatants from Mcat+NTHI parent or Mcat+ NTHI complemented Δ*luxS* biofilms exposed to anti-rsPilA. These results supported a role for NTHI quorum signaling via the LuxS pathway that, in addition to NTHI, also resulted in dispersal of Mcat from a dual-species biofilm.

10.1128/mBio.02423-18.4 FIG S4 Anti-rsPilA-mediated dispersal of NTHI+Mcat biofilms was dependent on NTHI Δ*luxS*. Dual-species biofilms established for 16 h at 37°C were exposed to anti-rsPilA or naive serum for 6 h. Anti-rsPilA exposure significantly dispersed both NTHI (A) and Mcat (B) from biofilms formed by Mcat+NTHI parent strain. In contrast, neither NTHI nor Mcat was dispersed from biofilms formed with Mcat+NTHI Δ*luxS*. Use of the complemented Δ*luxS* mutant to form dual-species biofilms restored the dispersal phenotype. Statistical significance was determined by one-way analysis of variance with the Holm-Sidak correction. *, *P ≤ *0.05; **, *P ≤ *0.01; ***, *P ≤ *0.001. Download FIG S4, TIF file, 3.0 MB. Copyright © 2018 Mokrzan et al. 2018 Mokrzan et al. This content is distributed under the terms of the Creative Commons Attribution 4.0 International license.

**FIG 6:**
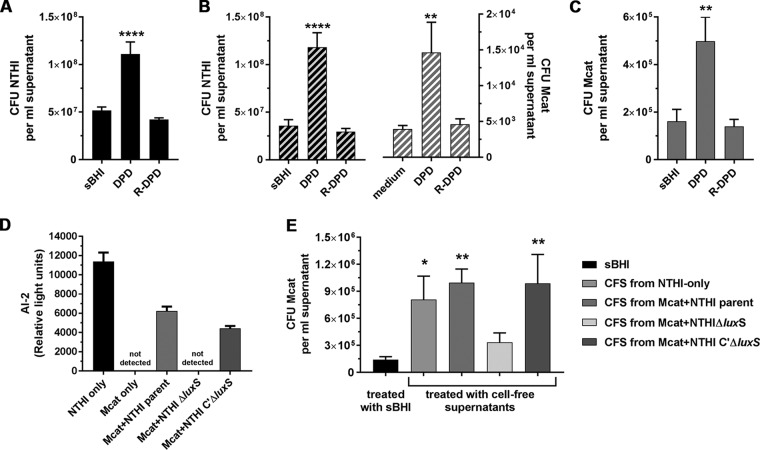
AI-2 produced by NTHI induced Mcat dispersion from dual-species biofilms. (A to C) NTHI+Mcat biofilms established at 37°C were exposed to medium, DPD, or R-DPD (negative control), and the dispersal of each bacterial species was enumerated. (A) DPD induced significantly greater dispersal of NTHI from monospecies biofilms compared to R-DPD or medium alone. (B) CFU of NTHI (black striped bars) and Mcat (gray striped bars) were significantly greater in supernatants collected from 16-h NTHI+Mcat biofilms exposed to DPD, compared to R-DPD. (C) CFU of Mcat were significantly greater in supernatants taken from 24-h monospecies biofilms treated with DPD, but not R-DPD. (D) Biofilms formed at 37°C for 16 h were exposed to anti-rsPilA for 6 h at 37°C. AI-2 was present in cell-free supernatants (CFS) from biofilms formed by NTHI only, Mcat+NTHI parent strain, or Mcat+complemented Δ*luxS* strain, but not by Mcat-only or Mcat+NTHI Δ*luxS*. (E) Mcat*-*only biofilms established for 24 h were exposed to sBHI or CFS from biofilms formed by NTHI only, or Mcat and either NTHI parent strain, Δ*luxS* strain, or complemented Δ*luxS* strain. As assessed by CFU/ml supernatant, Mcat was dispersed from a biofilm in response to CFS from NTHI-only or NTHI parent+Mcat biofilms, but not CFS from NTHI Δ*luxS*+Mcat biofilms. CFS from dual-species biofilms formed with the complemented Δ*luxS*+Mcat mutant restored Mcat dispersal. Together these results indicated that the anti-rsPilA-induced Mcat dispersal from NTHI+Mcat biofilms was mediated by AI-2 produced by NTHI in response to exposure to anti-rsPilA. Data represent mean ± SEM from 3 to 4 experiments performed in duplicate. Statistical significance was determined by one-way analysis of variance with the Holm-Sidak correction. Asterisk denotes significant difference from medium alone. *, *P ≤ *0.05; **, *P ≤ *0.01; ****, *P ≤ *0.0001.

Next, to demonstrate that both Mcat and NTHI responded to AI-2, we exposed established biofilms to chemically synthesized AI-2 [(*S*)-4,5-dihydroxy-2,3-pentanedione; DPD] at 0.2 µM, a concentration that disperses NTHI-only biofilms (10). Here, significantly more NTHI was dispersed from monospecies biofilms exposed to DPD versus the biologically inactive stereoisomer R-DPD or medium, based on both optical density (Fig. S5A) and bacterial counts (Fig. 6A). DPD also caused significant dispersion of both NTHI and Mcat from dual-species biofilms compared to R-DPD or medium alone (Fig. 6B and Fig. S5B). To confirm the direct effect of AI-2 on Mcat, we exposed Mcat-only biofilms to these same agents. Both measurements of OD_490_ of supernatants and enumeration of CFU within supernatants indicated that significantly greater numbers of Mcat were released from biofilms exposed to DPD versus R-DPD or medium (Fig. 6C and Fig. S5C). Taken together, these results demonstrated the dispersal of Mcat from NTHI+Mcat biofilms in response to AI-2.

10.1128/mBio.02423-18.5 FIG S5 DPD induced dispersion of NTHI and Mcat from single- and dual-species biofilms. Biofilms formed at 37°C were exposed to medium alone or to 4,5-dihydroxy-2,3 pentanedione (DPD, chemically synthesized AI-2) at 0.2 µM, and OD_490_ of the supernatant was measured over time to detect bacterial dispersion. (A) 16-h NTHI-only biofilms dispersed after approximately 105 min of incubation with DPD. (B) 16-h NTHI+Mcat biofilms dispersed after approximately 75 min of DPD exposure. (C) 24-h Mcat-only biofilms dispersed after approximately 60 min of incubation with DPD. Download FIG S5, TIF file, 5.1 MB. Copyright © 2018 Mokrzan et al. 2018 Mokrzan et al. This content is distributed under the terms of the Creative Commons Attribution 4.0 International license.

With the knowledge that exposure to AI-2 was sufficient to disperse both NTHI and Mcat from a dual-species biofilm, we next examined the relative amounts of AI-2 released from biofilms after exposure to anti-rsPilA. We hypothesized that anti-rsPilA would interact specifically with the NTHI in the dual-species biofilms to induce the production of AI-2. Biofilms were established for 16 h with NTHI alone or Mcat cocultured with NTHI parent strain, *luxS* mutant, or complemented *luxS* mutant. After exposure to anti-rsPilA for 6 h, supernatants were collected and filtered to remove bacteria and the relative amount of AI-2 was determined by Vibrio harveyi luminescence assay. As expected, AI-2 was present in cell-free supernatants (CFS) collected from NTHI-only biofilms and dual-species biofilms formed with Mcat+NTHI parent strain; however, AI-2 was not detected in CFS from Mcat-only or Mcat+NTHI Δ*luxS* biofilms (Fig. 6D). AI-2 levels in CFS from Mcat+complemented NTHI *Δlux*S were similar to those of Mcat+NTHI parent CFS. Interestingly, a significant amount of AI-2 was also produced when planktonic cultures of NTHI were incubated with anti-rsPilA (Fig. S6). This observation indicated that anti-rsPilA as an immune stressor induces a quorum-signaling response in NTHI irrespective of planktonic or biofilm-resident status. For both planktonic and biofilm-resident NTHI, AI-2 levels were significantly greater after exposure to anti-rsPilA versus naive serum or medium alone (Fig. S6 and S7).

10.1128/mBio.02423-18.6 FIG S6 Exposure to anti-rsPilA induced expression of AI-2 in planktonically grown NTHI. NTHI was suspended at an OD_490_ of 0.65, diluted 1/6 in sBHI, and incubated for 3 h at 37°C with 5% CO_2_ to induce Tfp expression (6). Two-hundred-microliter aliquots were exposed to 11 µg of IgG-enriched anti-rsPilA or naive serum for 6 h, filtered to remove bacteria, and frozen for subsequent AI-2 quantitation. NTHI cultures exposed to anti-rsPilA contained significantly more AI-2 than cultures exposed to medium alone. Exposure to naive serum did not affect AI-2 levels. Data represent mean ± SEM from 3 experiments performed in duplicate. Statistical significance was determined by Student’s *t* test. **, *P ≤ *0.01. Download FIG S6, TIF file, 1.0 MB. Copyright © 2018 Mokrzan et al. 2018 Mokrzan et al. This content is distributed under the terms of the Creative Commons Attribution 4.0 International license.

10.1128/mBio.02423-18.7 FIG S7 NTHI+Mcat biofilms produced more AI-2 after exposure to anti-rsPilA compared to naive serum. NTHI+Mcat biofilms were established for 16 h at 37°C and then exposed to sBHI or to 11 µg/well of either anti-rsPilA or naive serum IgG. Levels of AI-2 were significantly greater in CFS from biofilms exposed to anti-rsPilA versus naive serum or sBHI. Data represent mean ± SEM from 3 experiments performed in duplicate. Statistical significance was determined by one-way analysis of variance with the Holm-Sidak correction. **, *P ≤ *0.01; ***, *P ≤ *0.001. Download FIG S7, TIF file, 1.0 MB. Copyright © 2018 Mokrzan et al. 2018 Mokrzan et al. This content is distributed under the terms of the Creative Commons Attribution 4.0 International license.

To demonstrate directly that Mcat was dispersed in response to AI-2 produced by NTHI specifically, 24-h Mcat-only biofilms were incubated with CFS recovered from NTHI-only, NTHI parent+Mcat, NTHI Δ*luxS*+Mcat, or NTHI complemented Δ*luxS*+Mcat biofilms after exposure to anti-rsPilA. After 60 min (the time required for DPD-induced dispersion; see Fig. S5C), we quantitated the Mcat dispersed into the supernatants. Compared to biofilms maintained in medium alone, significantly more Mcat bacteria were detected in supernatants of Mcat-only biofilms exposed to CFS from biofilms in which LuxS was functional (Fig. 6E). In contrast, there was no dispersion of Mcat from monospecies biofilms after exposure to CFS from Mcat+NTHI Δ*luxS* biofilms, consistent with the lack of AI-2 detected in these CFS. Moreover, CFS from complemented Mcat+NTHI Δ*luxS* biofilms induced Mcat dispersion similar to that seen with CFS from Mcat+NTHI parent. Collectively, these results demonstrated that AI-2 produced by NTHI in response to anti-rsPilA induced the dispersion of Mcat from NTHI+Mcat biofilms.

### Antibiotic sensitivity of newly released bacteria equaled or surpassed that of their planktonic counterparts.

Our results thus far showed that antisera against rsPilA disrupted NTHI+ Mcat biofilms by active dispersal of both bacterial species. This dispersal has important biological consequences, as we and others have shown that bacteria newly released from a biofilm are often manyfold more sensitive to killing by antibiotics than their biofilm-resident counterparts (24–26), with greater specificity shown in some cases to certain classes of antibiotics (27).

To evaluate the antibiotic sensitivity of newly released NTHI and Mcat, we exposed NTHI+Mcat biofilms to anti-rsPilA, collected the newly released bacteria, and then incubated this subpopulation with antibiotics commonly used to treat respiratory tract diseases due to NTHI. For comparison, we tested the same antibiotics on a broth suspension of NTHI and Mcat that recapitulated the bacterial composition of the newly released populations (Fig. 5B) in terms of relative concentrations of each bacterial species, designated here “mixed suspension.” Additionally, we treated NTHI and Mcat resident within a dual-species biofilm with the same antibiotic concentrations, indicated as “biofilm resident.”

**FIG 7:**
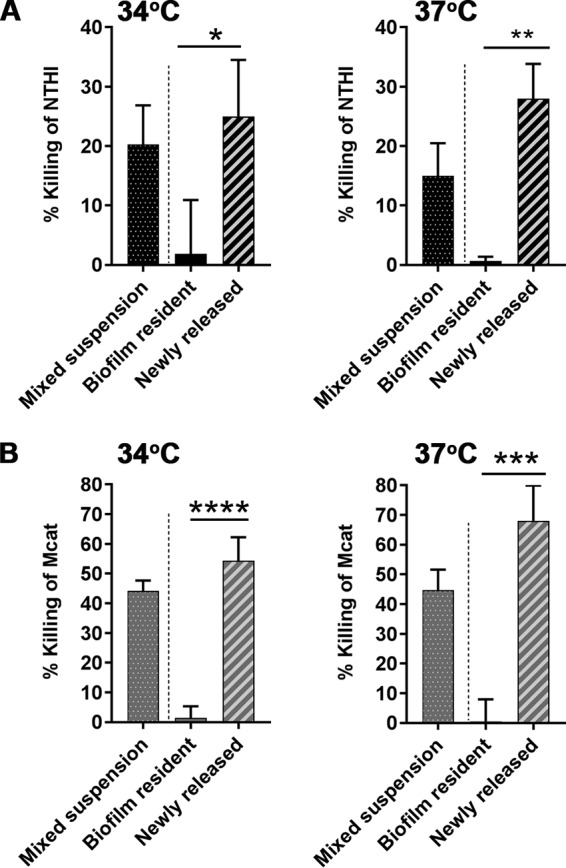
NTHI or Mcat newly released from dual-species biofilms was more sensitive to amoxicillin-clavulanate than biofilm-resident bacteria. Percent killing of NTHI or Mcat from mixed bacterial suspensions (dotted bars), biofilm-resident bacteria (solid bars), or newly released bacteria (striped bars) exposed to amoxicillin-clavulanate for 2 h at 34°C or 37°C. (A) The percent killing of newly released NTHI by 1.0/0.5 µg amoxicillin-clavulanate per ml was significantly greater than that of the biofilm-resident population at 34°C or 37°C. (B) The percent killing of newly released Mcat by 1.0/0.5 µg amoxicillin-clavulanate per ml (at 34°C) or 2.5/1.25 µg amoxicillin-clavulanate per ml (at 37°C) was significantly greater than that of the biofilm-resident population at either temperature. Data represent mean ± SEM from 3 experiments performed in duplicate. Statistical significance was determined by one-way analysis of variance with the Holm-Sidak correction. *, *P ≤ *0.05; **, *P ≤ *0.01; ***, *P ≤ *0.001; ****, *P ≤ *0.0001.

Amoxicillin-clavulanate is one of the most commonly prescribed antibiotics for OM, rhinosinusitis, and exacerbations of cystic fibrosis. Amoxicillin inhibits bacterial cell wall synthesis, and clavulanate enhances this effect by inhibition of bacterial beta-lactamases. Therefore, we utilized this antibiotic combination to test the antibiotic sensitivity of the mixed suspension, biofilm-resident, or newly released populations. NTHI in a mixed suspension was sensitive to killing by amoxicillin-clavulanate per ml, as shown by 20% ± 8% killing at 34°C and 15% ± 5% killing at 37°C (Fig. 7A). In contrast, NTHI within an NTHI+Mcat biofilm was not killed by this antibiotic combination at either temperature. Importantly, in the newly released population, we observed NTHI killing of 25% ± 9% at 34°C or 28% ± 6% at 37°C. These results indicated that newly released NTHI was significantly more sensitive to amoxicillin-clavulanate than biofilm-resident NTHI, and at least as sensitive as broth-suspended counterparts, at either temperature tested.

Likewise, Mcat in a mixed suspension was also susceptible to amoxicillin-clavulanate, as shown by 44% ± 8% killing or 45% ± 7% killing at 34°C or 37°C, respectively (Fig. 7B). As expected, Mcat resident within an NTHI+Mcat biofilm was not killed at either temperature. However, Mcat newly released from a dual-species biofilm by the action of anti-rsPilA was highly sensitive to amoxicillin-clavulanate, with 54% ± 8% killing at 34°C or 68% ± 12% killing at 37°C. The percent killing in the newly released population was significantly greater than that of biofilm-resident Mcat, and at least as sensitive as broth-suspended Mcat (Fig. 7B). These results showed that whereas amoxicillin-clavulanate had no effect on biofilm-resident NTHI or Mcat, bacteria newly released from the biofilm by exposure to anti-rsPilA were significantly more sensitive to killing by this first-line antibiotic.

**FIG 8:**
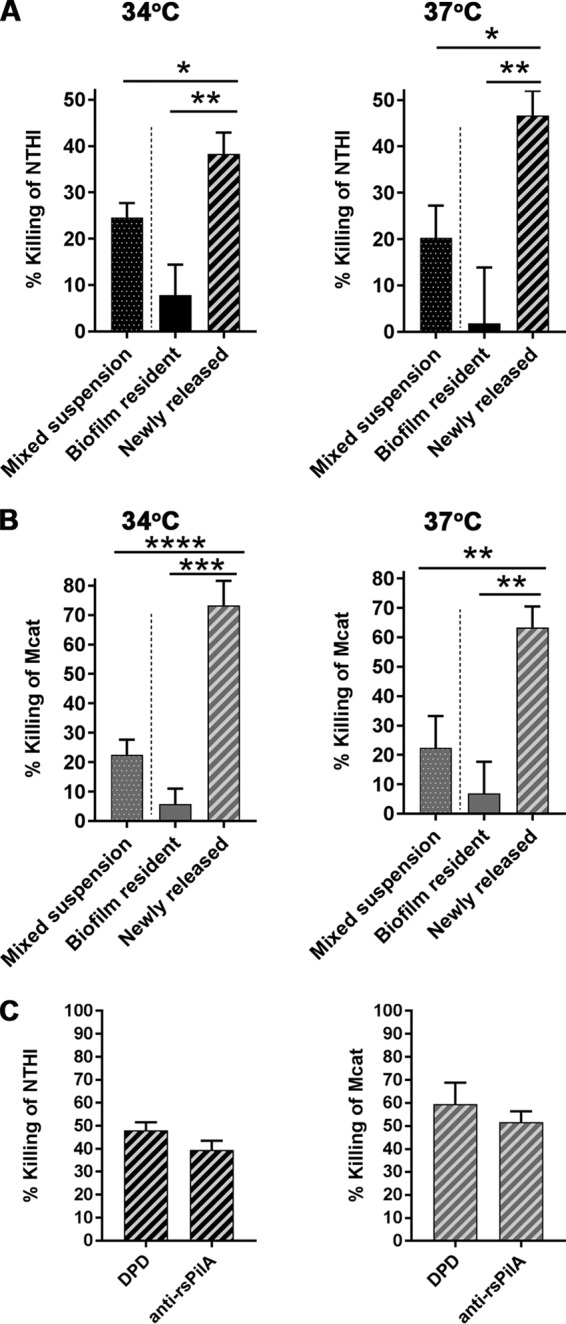
Newly released NTHI or Mcat was highly sensitive to antibiotics that inhibit protein synthesis. Percent killing of NTHI or Mcat from mixed bacterial suspensions (dotted bars), biofilm-resident bacteria (solid bars), or newly released bacteria (striped bars) exposed to antibiotics for 2 h at 34°C or 37°C. (A) Newly released NTHI was significantly more sensitive to 7.5/37.5 µg trimethoprim-sulfamethoxazole per ml than either biofilm-resident NTHI or NTHI in a mixed suspension, regardless of temperature. (B) Newly released Mcat was significantly more sensitive to 20 µg clarithromycin per ml than either biofilm-resident Mcat or planktonic Mcat in a mixed suspension, at 34°C or 37°C. (C) NTHI (black striped bars) and Mcat (gray striped bars) dispersed from a dual-species biofilm by DPD versus anti-rsPilA were equally sensitive to killing by trimethoprim-sulfamethoxazole at 7.5/37.5 µg/ml (NTHI) or clarithromycin at 20 µg/ml (Mcat). Data represent mean ± SEM from 3 experiments performed in duplicate. Statistical significance was determined by one-way analysis of variance with the Holm-Sidak correction. *, *P ≤ *0.05; **, *P ≤ *0.01; ***, *P ≤ *0.001; ****, *P ≤ *0.0001. These data suggested that the dispersal process mediated by anti-rsPilA enhanced the sensitivity of both NTHI and Mcat to killing as mediated via inhibition of protein synthesis.

A previous report showed that the antibiotic sensitivity of newly released bacteria varied depending upon the antibiotic class (27). Therefore, here we examined the relative sensitivity of suspended, biofilm-resident, or newly released NTHI and Mcat to antibiotics that inhibit protein synthesis. We chose a combination of trimethoprim-sulfamethoxazole to use against the NTHI in our assays, since this combination is indicated for use against NTHI clinically (28, 29). Treatment with trimethoprim-sulfamethoxazole killed 24% ± 3% or 20% ± 7% of NTHI in a mixed suspension at 34°C or 37°C, respectively (Fig. 8A). As anticipated, NTHI resident within an NTHI+Mcat biofilm was not readily killed by this antibiotic concentration at either temperature. However, newly released NTHI was highly sensitive to trimethoprim-sulfamethoxazole, with 38% ± 5% killing at 34°C and 45% ± 7% killing at 37°C. Not only were these values significantly greater than those observed for NTHI resident within a biofilm, but they were also significantly greater than the values for NTHI in a mixed suspension. Furthermore, this significantly greater antibiotic sensitivity was not due to toxicity of anti-rsPilA itself, since the addition of anti-rsPilA had no effect on the antibiotic sensitivity of NTHI in a mixed suspension (Fig. S8A to D).

10.1128/mBio.02423-18.8 FIG S8 Anti-rsPilA did not affect antibiotic sensitivity of NTHI or Mcat in a mixed suspension. CFU of NTHI (A to D) or Mcat (E to H) per ml in mixed suspensions exposed to the indicated antibiotics or medium alone for 2 h at 34°C or 37°C. Exposure to anti-rsPilA (striped bars) had no significant effect on NTHI killing by amoxicillin-clavulanate (A and C) or trimethoprim-sulfamethoxazole (B and D) at either 34°C or 37°C. Likewise, exposure to anti-rsPilA did not significantly affect Mcat killing by amoxicillin-clavulanate (E and G) or clarithromycin (F and H) at either 34°C or 37°C. Data represent mean ± SEM from 3 experiments performed in duplicate. Statistical significance was determined by Student’s *t* test. Download FIG S8, TIF file, 10.5 MB. Copyright © 2018 Mokrzan et al. 2018 Mokrzan et al. This content is distributed under the terms of the Creative Commons Attribution 4.0 International license.

To similarly evaluate the sensitivity of newly released Mcat to killing by an antibiotic that acted via protein inhibition, we chose clarithromycin, which has greater activity against Mcat than against NTHI (30, 31). Treatment with clarithromycin killed 22% ± 5% and 22% ± 11% of Mcat in a mixed suspension at 34°C and 37°C, respectively (Fig. 8B). As expected, Mcat that was resident within an NTHI+Mcat biofilm was not readily killed by this antibiotic at either temperature. However, newly released Mcat was markedly more sensitive to clarithromycin, with 73% ± 8% killing at 34°C and 63% ± 7% killing at 37°C. This percentage of killing was significantly greater than that of Mcat when resident within an NTHI+Mcat biofilm as well as Mcat within a mixed suspension. Again, these results were not due to anti-rsPilA itself, since the antibiotic sensitivity of Mcat within a mixed suspension was not affected by anti-rsPilA (Fig. S8E to H).

Taken together, these results demonstrated that anti-rsPilA-mediated dispersal from a dual-species biofilm significantly enhanced the sensitivity of both the newly released NTHI and Mcat to several classes of antibiotics, with preferential enhanced sensitivity to those that work through inhibition of protein synthesis. We wondered whether this enhanced sensitivity to antibiotics was completely mediated by release from the biofilm due to the AI-2 quorum signaling process, or whether anti-rsPilA *per se* might mediate an additional effect. Accordingly, we compared the antibiotic sensitivities of NTHI and Mcat that were newly released from a dual-species biofilm by anti-rsPilA or AI-2 (DPD). We reasoned that, if anti-rsPilA influenced antibiotic sensitivity in multiple ways, then NTHI or Mcat dispersed from a dual-species biofilm by the action of DPD alone would be less sensitive to antibiotic killing than their counterparts that had been dispersed via the actions of anti-rsPilA. We found that NTHI dispersed via DPD or anti-rsPilA displayed similar sensitivity to killing by trimethoprim-sulfamethoxazole (Fig. 8C). Similarly, Mcat dispersed from an NTHI+Mcat biofilm via exposure to DPD or anti-rsPilA also showed similar sensitivity to clarithromycin. We conclude that AI-2-induced dispersal of NTHI and Mcat from a dual-species biofilm was sufficient to recapitulate the enhanced antibiotic sensitivity phenotype observed in NTHI and Mcat that were newly dispersed from dual-species biofilms by anti-rsPilA.

## DISCUSSION

The candidate vaccine antigen rsPilA targets the major subunit of NTHI Tfp and is highly effective as an immunogen for both the prevention and the therapeutic resolution of NTHI-induced otitis media in experimental models (11, 12). Epidemiological studies that examine middle ear fluids taken from patients with OM show that NTHI is frequently isolated together with Mcat (13, 14). Consequently, strategies to prevent or treat OM due to NTHI alone, as well as to treat disease due to combinations of NTHI and additional bacterial species such as Mcat, are greatly needed. The chronic and recurrent nature of OM is due to the presence of bacterial biofilms in the middle ear, and we have shown that antisera that target the majority subunit of NTHI Tfp can both inhibit NTHI biofilm formation and disrupt an established NTHI biofilm *in vitro* (9, 10) and *in vivo* (11, 12). In the present study, we tested the ability of anti-rsPilA to inhibit the formation of dual-species biofilms formed by NTHI and Mcat as well as disrupt established dual-species biofilms.

Expression of PilA, the major subunit of the NTHI Tfp, is tightly regulated and influenced by environmental conditions, including pH (5) and even the subtle three-degree temperature difference between the site of asymptomatic colonization, the human nasopharynx (34°C), and the site of infection during OM, the middle ear (37°C) (9). The nasopharynx is a dynamic environment with mechanical stresses from airflow, ciliary activity, and liquid/mucus movement. In both the nasopharynx and middle ear, NTHI Tfp are important to mediate adherence to epithelial surfaces as well as to other bacteria (5–8) and for biofilm formation, among many other important biological roles. Thus, the accelerated expression of NTHI Tfp at 34°C as NTHI+Mcat biofilms form, as shown here, likely contributes to the persistence of both species in the nasopharynx.

Early exposure of NTHI+Mcat biofilms to anti-rsPilA, anti-OMP P5, or anti-chimV4 inhibited biofilm formation, with a clear additive effect when both of these NTHI adhesins were targeted (e.g., via the use of anti-chimV4). These results suggested that immunization with rsPilA or chimV4 will likely limit the formation of dual-species biofilms *in vivo* in both the nasopharynx and the middle ear. It is important to note that the concentration of antiserum used in these studies was arbitrarily chosen, and although it was significantly effective, it likely underrepresented the maximal benefit that could be obtained by active immunization.

With the knowledge that anti-rsPilA significantly disrupts established NTHI-only biofilms (9, 10), here we showed that anti-rsPilA can also significantly disrupt a dual-species biofilm with dispersal of both NTHI and Mcat from these biofilms. The ability of antibodies that specifically target a single NTHI antigen to disperse a second predominant pathogen of OM (that did not express this antigen) was unexpected and intriguing as our data strongly suggested that immunization with rsPilA was likely to engender an important collateral benefit when used in a therapeutic vaccine strategy.

Quorum-sensing signals coordinate group activities among bacterial communities. The *luxS* quorum sensing system is highly conserved among many bacterial species. AI-2 is considered to be an interspecies quorum signal (32, 33) because some bacteria that do not produce AI-2 themselves are nonetheless able to “eavesdrop” on quorum-sensing signals from other species (34, 35). Armbruster et al. (19) demonstrated that during early biofilm formation *in vitro*, Mcat responds to exogenous AI-2 by producing biofilms with increased biomass and antibiotic resistance. Here we demonstrated that mature biofilms formed by NTHI, Mcat, or NTHI+Mcat responded to exogenous AI-2 with a dispersal event (Fig. 6; see also Fig. S5 in the supplemental material). We concluded that NTHI resident in a mature biofilm responds differently to the quorum signal AI-2 than does NTHI that is planktonic or in the early stages of biofilm formation, and furthermore, in NTHI+Mcat biofilms this response is recapitulated by Mcat. Although the amount of AI-2 detected in supernatants from NTHI-only biofilms exposed to anti-rsPilA was nearly twice that detected in supernatants from similarly exposed NTHI+Mcat biofilms, CFS from NTHI-only or dual-species biofilms induced a similar degree of Mcat dispersal from Mcat-only biofilms (Fig. 6E). We therefore speculate that there is a threshold level of AI-2 above which biofilm dispersal is induced.

There is a growing body of evidence that bacteria newly released from a biofilm are transcriptionally and phenotypically distinct from either biofilm-resident or planktonically grown bacteria (36–39). Several reports have shown a heightened sensitivity to antibiotic killing of newly released NTHI, Burkholderia cenocepacia, and Staphylococcus epidermidis, in some cases even greater than that of planktonic bacteria (24–26). However, Chambers et al. (27) showed that the antibiotic sensitivity of newly released Pseudomonas aeruginosa differed with the mode of dispersal and the antibiotic tested. In the present study, we found that after dispersal from a dual-species biofilm mediated by anti-rsPilA, both NTHI and Mcat became significantly more susceptible to amoxicillin-clavulanate than their biofilm-resident counterparts. Moreover, and importantly, newly released NTHI and Mcat were both significantly more sensitive to killing by antibiotics that inhibit protein synthesis than their biofilm-resident counterparts as well as their counterparts in a mixed suspension. Interestingly, Chambers and coworkers (27) also found that P. aeruginosa dispersed by glutamate became highly sensitive to tobramycin, which also works through inhibition of protein synthesis. Quorum sensing is known to induce widespread metabolic changes in many bacterial species (40, 41). We speculate that AI-2-mediated dispersal induced by anti-rsPilA exposure may cause metabolic changes that render both NTHI and Mcat highly sensitive to antibiotics. In support of this notion, NTHI and Mcat that were dispersed from dual-species biofilms by either exogenous AI-2 (DPD) or exposure to anti-rsPilA displayed similar heightened sensitivity to antibiotics that inhibit protein synthesis (Fig. 8C). Alternatively, the increased sensitivity of newly dispersed NTHI and Mcat to antibiotics that inhibit protein synthesis may reflect changes in metabolism or transcription due to the newly dispersed state of NTHI and Mcat, compared to the biofilm-resident state, the latter of which is generally considered to be metabolically quiescent (42). Although these newly dispersed bacteria are significantly more sensitive to antibiotics than their biofilm-resident counterparts, additional work is necessary to fully understand the mechanisms behind this increased sensitivity (40, 41).

For OM due to NTHI or Mcat, amoxicillin-clavulanate is a common first-line antibiotic of choice for treatment. However, the results of our study offer insights into the development of novel treatment paradigms for OM. Combination of vaccine-induced biofilm dispersal with the killing action of traditional antibiotics that inhibit protein synthesis could exploit the newly released bacterial phenotype, and thereby greatly reduce the dose of antibiotic required to cure disease. Moreover, as NTHI and Mcat have been detected together in the lungs of children with cystic fibrosis (43) and patients with chronic obstructive pulmonary disease (44), the strategy of immunization with rsPilA combined with a lower dose of appropriate antibiotics represents a novel approach to the treatment of multiple chronic and recalcitrant biofilm-associated diseases.

In summary, we have demonstrated that antisera against the majority subunit of NTHI Tfp, PilA, inhibited the formation of dual-species NTHI+Mcat biofilms. In addition, antiserum against rsPilA disrupted preestablished NTHI+Mcat biofilms by dispersing not only NTHI but also Mcat from these biofilms. Furthermore, newly released NTHI and Mcat were highly sensitive to traditional antibiotics that are otherwise ineffective when these bacteria reside within a biofilm, with enhanced sensitivity to those that specifically inhibited protein synthesis. These results expand the utility of the vaccine candidate rsPilA to include use against polymicrobial OM due to NTHI and Mcat and offer a new potentially powerful defense against the growing threat of antibiotic resistance by combining the power of active immunization with the killing activity of traditional antibiotics that could be used at a reduced dose.

## MATERIALS AND METHODS

### Ethics statement.

Nontypeable Haemophilus influenzae 86-028NP and Moraxella catarrhalis 7169 are archived strains originally described in earlier publications (45, 46). Both strains have been anonymized.

### Bacterial strains and biofilms.

Strains used are shown in Table 1. Bacteria were grown on chocolate agar or in brain heart infusion broth supplemented with 2 µg each of heme and β-NAD per ml (sBHI). Biofilms were established as previously described (47). For dual-species biofilms, NTHI and Mcat were inoculated together at a ratio of either 1:1 or 1:3, with a total of 4 × 10^4^ bacteria per well, and incubated in 5% CO_2_-air at either 37°C or 34°C. After 24 h in culture, NTHI was about 100-fold more numerous than the Mcat in dual-species biofilms; to prevent Mcat from being outcompeted by the faster-growing NTHI in our culture system, we increased this ratio to 1:3 NTHI to Mcat in all subsequent experiments. We enumerated CFU of NTHI or Mcat by serial dilution and plating on chocolate agar with either 10 µg vancomycin and 5 µg trimethoprim per ml or 15 µg ampicillin per ml (Northeast Laboratories, Winslow, ME, USA), respectively (18).

**TABLE 1:** Bacterial strains used in this study

Bacterial strain	Description	Reference(s)
NTHI 86-028NP	Archived strain originally isolated from the nasopharynx of a child with chronic OM	45
M. catarrhalis 7169	Archived strain originally obtained via tympanocentesis from the middle ear of a child with OM	46
NTHI:*ompP2*-GFP	NTHI 86-028NP in which expression of GFP is under control of the *ompP2* promoter	51
NTHI:*pilA*-GFP	NTHI 86-028NP in which expression of GFP is under control of the *pilA* promoter	10
NTHI Δ*luxS*	NTHI 86-028NP *luxS* mutant generated by insertion of kanamycin resistance cassette within *luxS* gene	9, 52
NTHI Δ*luxS*/pSPEC1-*luxS*	Complemented NTHI 86-028NP *luxS* mutant	10, 51

### Biofilm visualization.

To distinguish between Mcat and NTHI, dual-species biofilms were seeded with (nonfluorescent) Mcat 7169 and NTHI:*ompP2*-GFP, which constitutively expresses GFP. After 16 h of incubation at 34°C or 37°C, biofilms were rinsed and stained with FM4-64 dye {*N*-(3-triethylammoniumpropyl)-4-(6-[4-(diethylamino)phenyl]hexatrienyl)pyridinium dibromide; Molecular Probes, Eugene, OR}, as described (9). Duplicate wells were imaged by confocal scanning laser microscopy (CSLM) with a Zeiss 510 Meta-laser microscope. For quantitation of biofilm metrics by COMSTAT2 analysis (48, 49), biofilms were stained with LIVE/DEAD BacLight Bacterial Viability kit (Molecular Probes) according to the manufacturer’s protocol and fixed overnight as previously described (9, 25). Images were rendered with Zeiss Zen software. Data represent the mean ± SEM.

### Scanning electron microscopy.

NTHI+Mcat biofilms were formed on 5-mm coverslips as described above for 24 h at 37°C and fixed overnight at 4°C in 2.5% glutaraldehyde. Biofilms were postfixed in osmium tetroxide (Electron Microscopy Sciences, Hatfield, PA) and dehydrated through graded alcohols. Dry coverslips were mounted onto 15-mm aluminum stubs, sputter coated with gold-palladium, and imaged on a Hitachi S-4800 scanning electron microscope.

### Biofilm inhibition and disruption assays.

Rabbit polyclonal antisera were generated at Spring Valley Laboratories (Woodbine, MD) and were not heat inactivated. Naive rabbit serum served as a negative control. Assays were performed as previously described (9). For biofilm inhibition assays, biofilms were exposed 1 h after seeding to an arbitrarily selected 1:50 dilution of antiserum. After 4 h, supernatants were replaced with fresh sBHI. After incubation overnight, biofilms were stained and visualized by CSLM. For biofilm disruption assays, biofilms were established for 24 h at 37°C or 34°C prior to treatment for 16 h with a 1:50 dilution of antiserum, as described previously (10, 25). Biofilms were stained and visualized, or else CFU of NTHI and/or Mcat were quantified as described above.

### Biofilm dispersal by DPD or CFS.

First, we determined the kinetics of mono- or dual-species biofilm dispersion by DPD (see Fig. S5 in the supplemental material). Biofilms were exposed to 0.2 µM DPD or R-DPD until the time of peak dispersion, and supernatants were diluted and plated to determine bacterial CFU. To assess Mcat dispersal by CFS, we first established NTHI+Mcat biofilms for 16 h at 37°C with Mcat and either NTHI parent, NTHI Δ*luxS,* or complemented NTHI Δ*luxS*. Biofilms were exposed to sBHI with 11 µg IgG-enriched anti-rsPilA for 6 h, and then supernatants were centrifuged at 16,000 × *g* for 5 min and sterile filtered. The resulting cell-free supernatant (CFS) was used immediately or stored at −80°C. Mcat*-*only biofilms were established for 24 h at 37°C, and then medium was replaced with 200 µl of fresh sBHI or CFS. After 60 min, supernatants were collected and plated for Mcat CFU.

### Detection of AI-2.

AI-2 concentration was quantified by a V. harveyi luminescence assay as previously described (10, 50).

### Antibiotic sensitivity of biofilm-resident, newly released, and mixed suspensions of NTHI and Mcat.

For the adherent population, NTHI+Mcat biofilms were established for 22 to 23 h at either temperature. Biofilms were washed twice and incubated in sBHI supplemented with the indicated antibiotics for 2 h. For the newly released population, NTHI+Mcat biofilms established for 16 h were exposed to IgG-enriched rabbit anti-rsPilA as described above. At the time of maximum bacterial dispersion (6 or 7 h for biofilms formed at 37°C or 34°C, respectively), supernatants were collected and incubated in sBHI supplemented with the indicated antibiotics for 2 h. For the mixed suspension, to replicate the numbers of NTHI and Mcat present in the newly released population after treatment with anti-rsPilA, NTHI and Mcat were suspended together in sBHI to a final concentration of 1.4E5 CFU Mcat and 5.9E8 CFU NTHI per ml as determined by colony counts. The mixed suspension was incubated in sBHI supplemented with the indicated antibiotics for 2 h. Exposure time was selected based on reports that after 2 h newly released bacteria begin to transition back to a planktonic phenotype (27, 39). CFU of NTHI or Mcat were determined by serial dilution and plating. Amoxicillin (Sigma-Aldrich, St. Louis, MO) and clavulanate (U.S. Pharmacopeia, Rockville, MD) were used at a ratio of 2:1. Trimethoprim (Sigma-Aldrich) and sulfamethoxazole (Santa Cruz Biotech, Dallas, TX) were used at a ratio of 1:5. Clarithromycin was obtained from TCI America (Portland, OR).

### Statistics.

All experiments were performed in duplicate with least 3 biological replicates, conducted on separate days. Data analyses were performed with GraphPad Prism software version 6. For multiple comparisons, one-way analysis of variance with the Holm-Sidak correction was used. All other comparisons were made with unpaired *t* tests.
