# Supplementary figures and images for: Antibodies against the Majority Subunit (PilA) of the Type IV Pilus of Nontypeable Haemophilus influenzae Disperse Moraxella catarrhalis from a Dual-Species Biofilm
Source: mBio. 2018 Dec 11;9(6):e02423-18. doi: 10.1128/mBio.02423-18 (PMC6299487; doi:10.1128/mBio.02423-18)

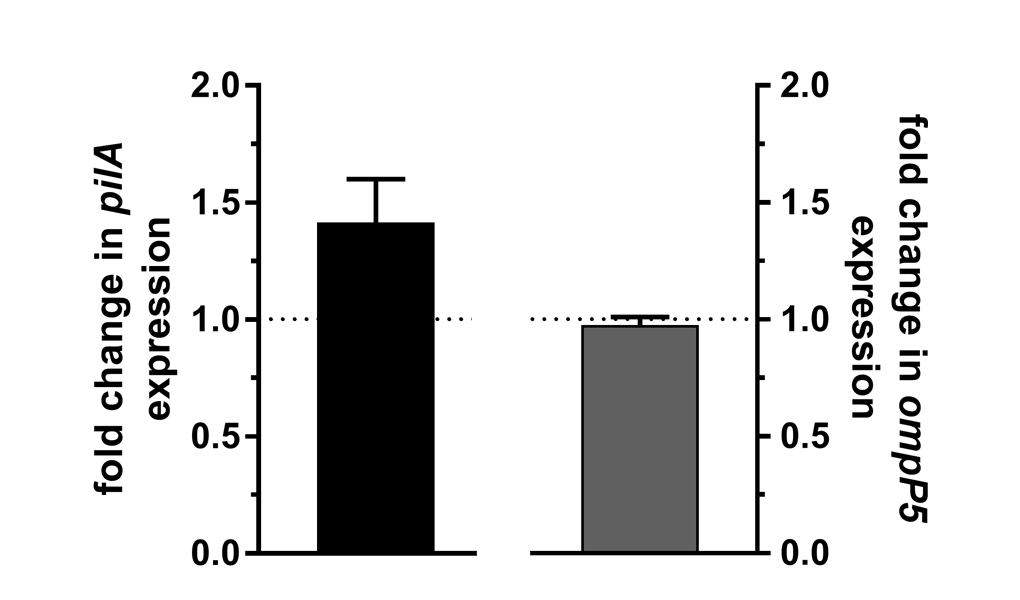

Supplement: FIG S1 [file mbo006184208sf1.tif]

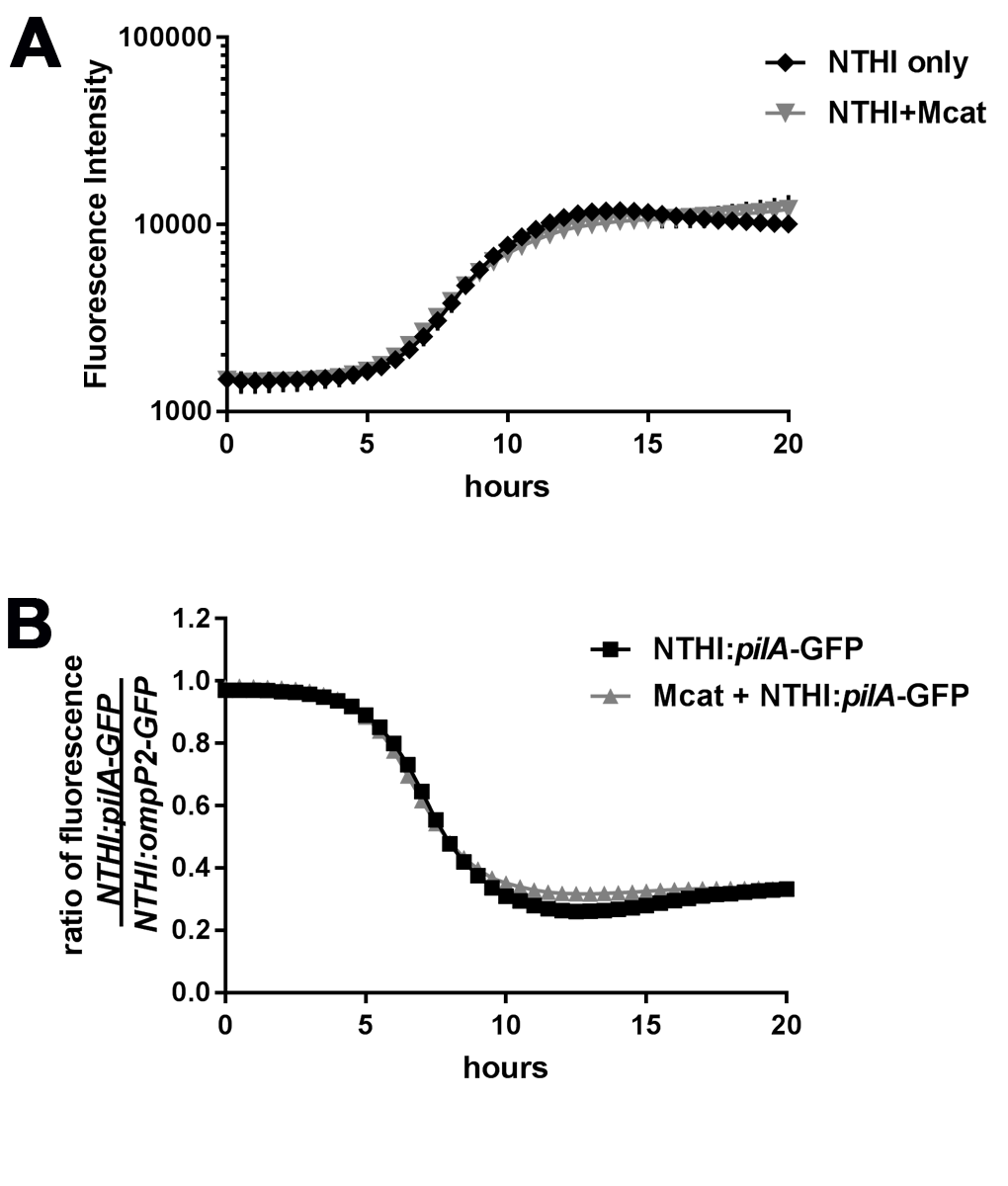

Supplement: FIG S2 [file mbo006184208sf2.tif]

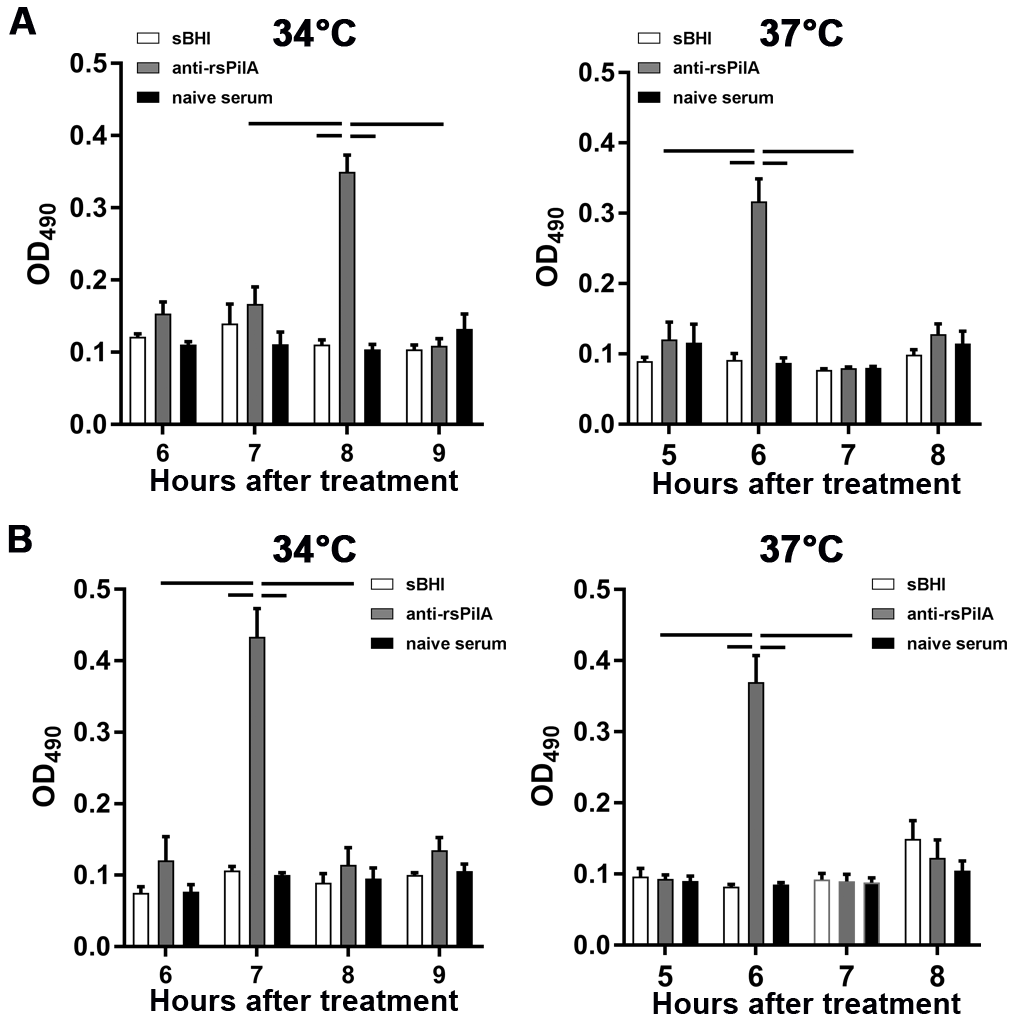

Supplement: FIG S3 [file mbo006184208sf3.tif]

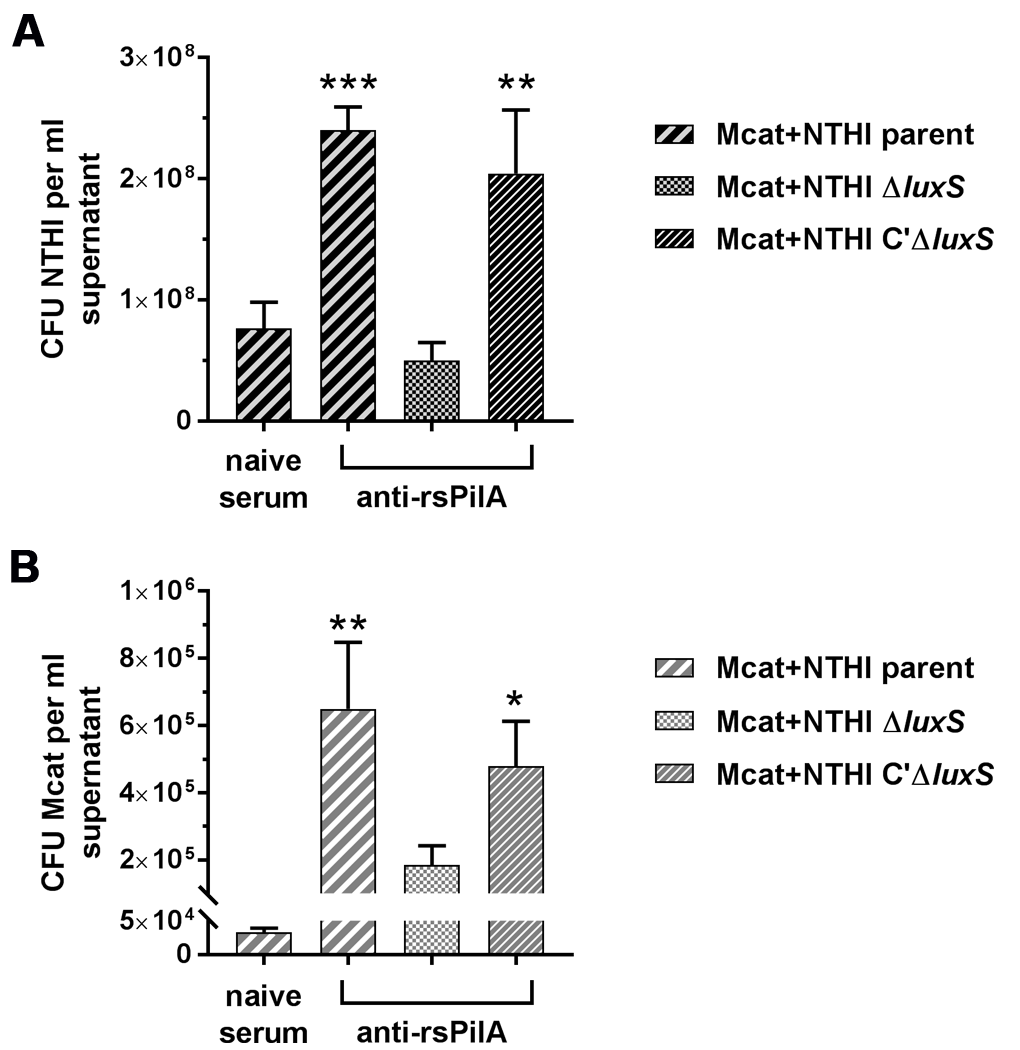

Supplement: FIG S4 [file mbo006184208sf4.tif]

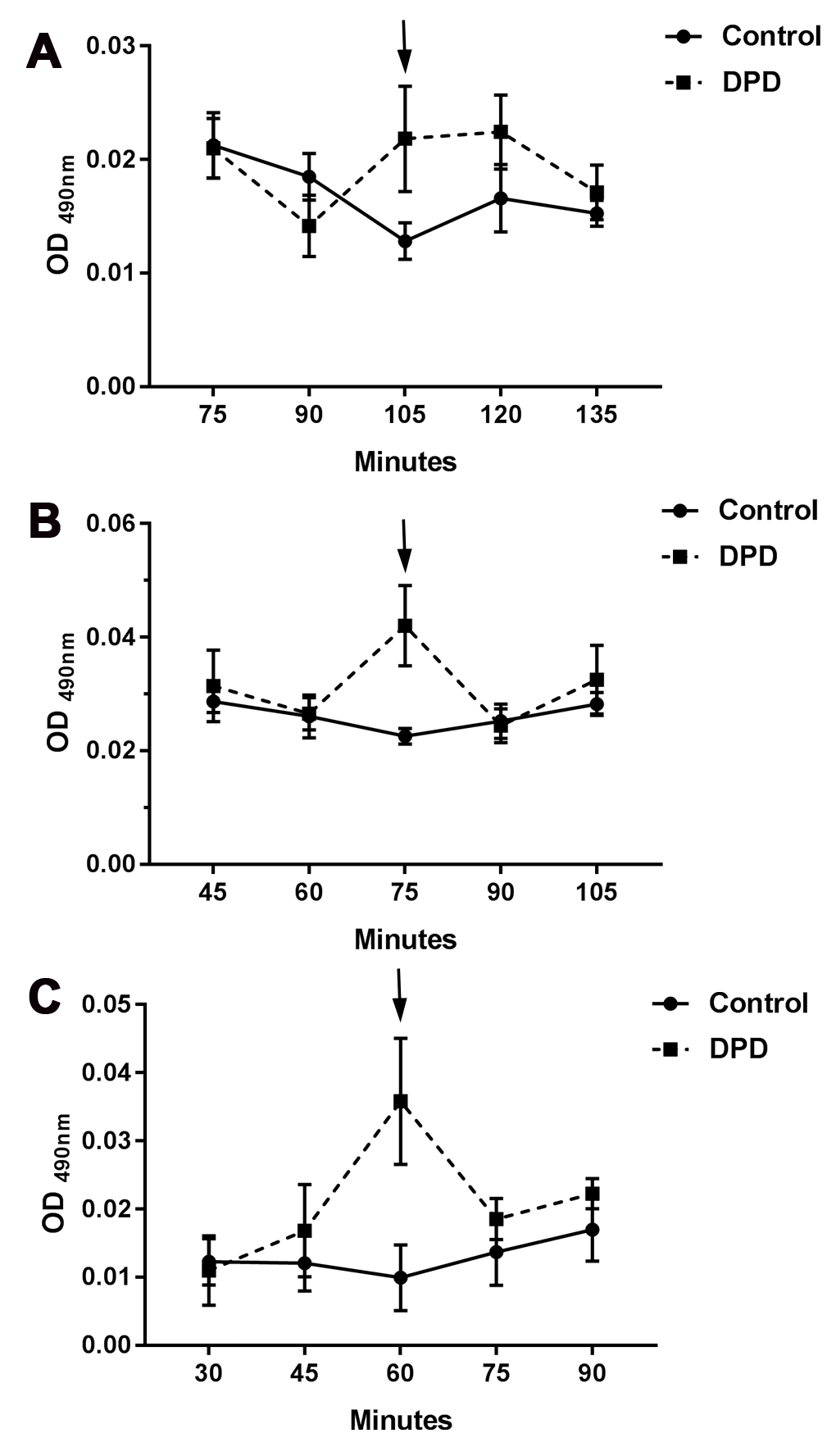

Supplement: FIG S5 [file mbo006184208sf5.tif]

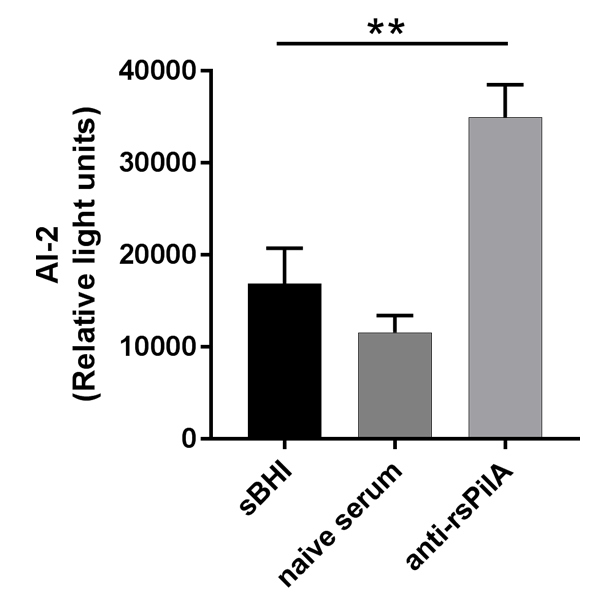

Supplement: FIG S6 [file mbo006184208sf6.tif]

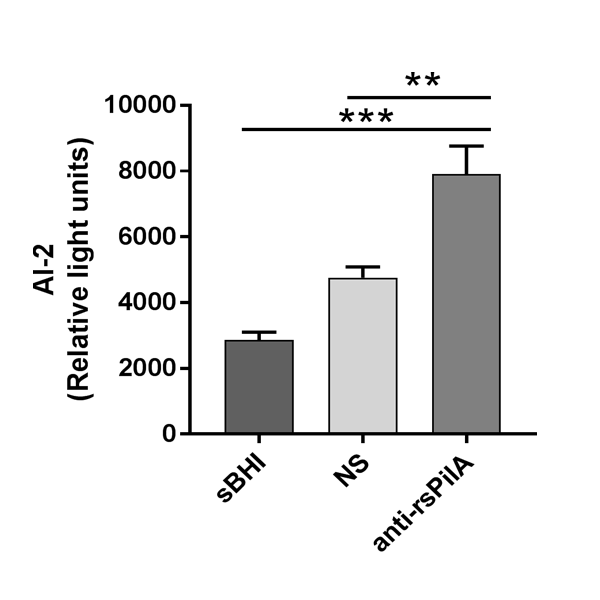

Supplement: FIG S7 [file mbo006184208sf7.tif]

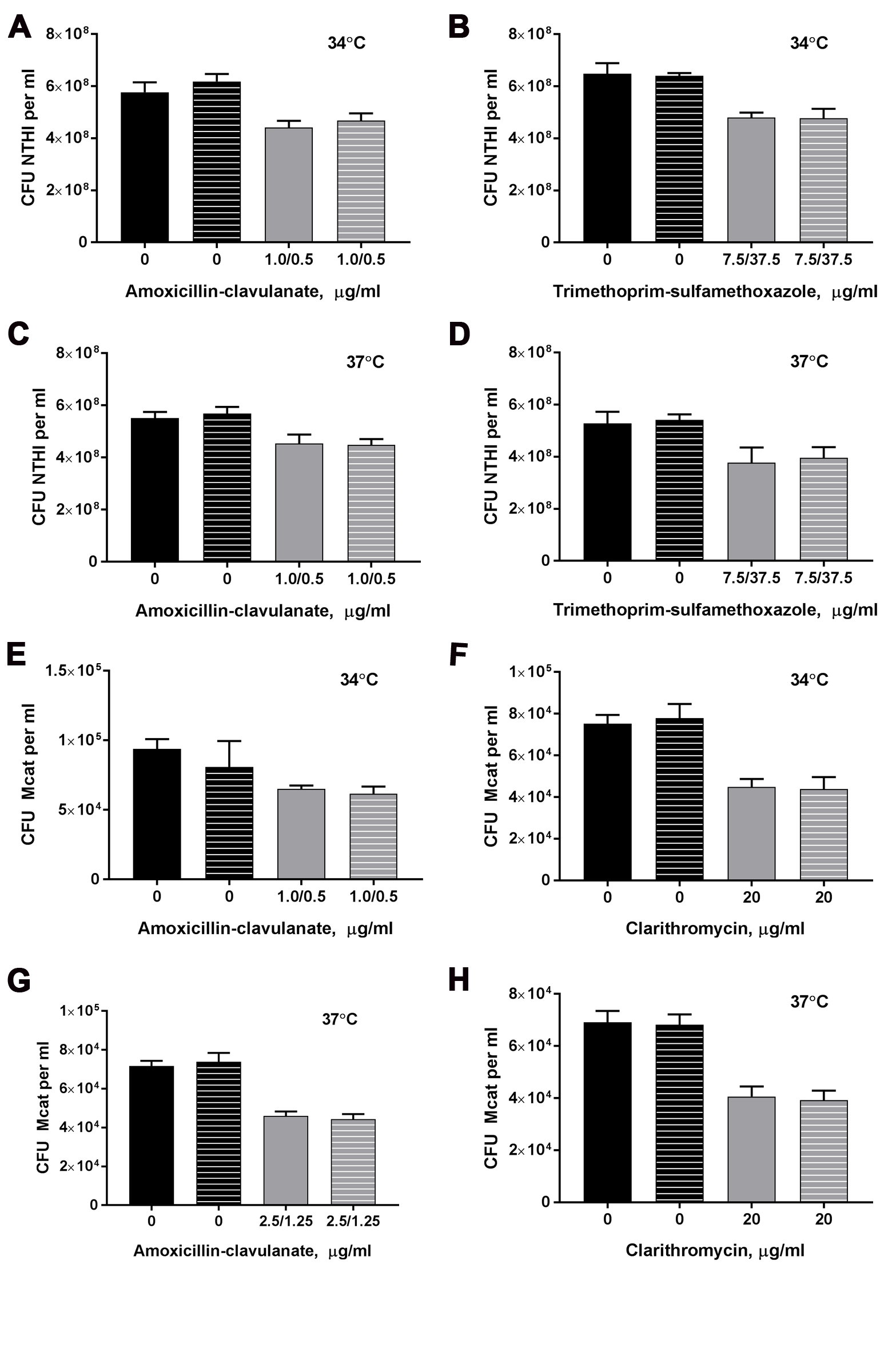

Supplement: FIG S8 [file mbo006184208sf8.tif]
